# Hospital Meetings, &c.

**Published:** 1900-06-16

**Authors:** 


### HOSPITAL MEETINGS, &C.

### LONDON HOSPITAL.

A quarterly general court of governors of the London Hospital was held on Wednesday, 6th inst. The Hon. Sydney Holland, chairman of the House Committee, presided, and read the report of the house governor to the committee for the past quarter.

The report stated that great progress had been made with the improvements which had been sanctioned. In the west forecourt the excavations had been made, the foundations laid, and the walls were now above the ground. This was for the enlargement of the receiving-room, and had already effected a noticeable change in the appearance of the hospital. In other parts also considerable progress had been made. The plans for the new out-patient department had been finally settled, and the work was to be pushed forward with the utmost expedition. Attention was called to the continued increase in the number of patients treated—the in-patients having risen from 10,559 in 1895 to 13,234 in 1899—and to the consequent advance in the cost of the upkeep of the hospital. An appeal was made to the governors to assist the committee in carrying out the idea that the hospital should be looked upon as a great consulting centre for dealing with all cases which were either obscure, difficult, or required expensive treatment. The report went on to state that the electric light had been introduced into some departments, and the current was also being used with great advantage for the driving of fans for ventilating various rooms. The tender of the National Telephone Company for the installation of a system of telephones throughout the hospital had been accepted, and the work was well forward. Two more houses had been taken and furnished, after the necessary repairs had been completed, in Newark Street for the nurses. The necessity for the increase of the accommodation for the nursing staff was very great, and now that the purchase of the John Baker's Almshouses had been completed the committee intended to prepare a scheme for building a nurses' home on this site at a cost of something like £20,000. It is two years since the decision to build such a home was come to. The entry of students at the college had again been a good one, and the medical school was in all respects in a flourishing condition.

The department for the light cure of lupus—technically known as photo-therapy—had just been opened under the charge of Dr. Stephan Mackenzie. The earliest experiences pointed to a very successful use of this mode of treatment. It had been determined that three sets of patients in batches of four in each set should be treated, each morning, free of charge, while the afternoon would be devoted to the treatment of two batches of patients, four in each batch, who could afford to pay for the treatment. The expense of the introduction of this mode of treatment had been considerable, but the number of patients who had been treated was very great, and without doubt an inestimable boon had been conferred on a large class of sufferers. It would be remembered that the Princess of Wales bore the whole cost of the first instruments—over £100. Mr. Openshaw, one of the visiting surgeons, had been called away, and was now on duty at the front. Special arrangements had been made for carrying on his work, and all was going on satisfactorily. The committee had very carefully considered the question of the payment of the men employed at the hospital. The number of porters was now considerable, and more was required of them, more labour being entailed by the system of carrying patients to the garden on all favourable occasions. It had been decided that the wages of all the porters working until ten p.m. on alternate nights should be raised 2s. per week, and a pension scheme had been introduced. The sub-committee appointed to select a site for the Herman de Stern Convalescent Home had chosen one at Felixstowe, which seemed in every way satisfactory. Negotiations for the purchase were making favourable progress. The home, when erected, is to be devoted entirely to the service of the London Hospital. It will cost £10,000 to build, and the executors have endowed it with £50,000. Special attention was called to the fact that on May 4th the total number of in-patients was 728 for the day, the highest record since 1876, when there were in one day 733 cases. It would be understood that it was most difficult to carry on the work with so many alterations going on, not the least of which was that everything had to be done with one lift. But the House Committee were determined to do their best to keep the hospital open during all the building operations. To do this, however, would cost £10,000 in temporary arrangements.

Mr. James Hora, a vice-president, who had already so generously helped the "Marie Celeste" Samaritan Society, had made a further donation of £2,000, and had promised £4,000 more. It was also announced with special gratification that the hospital had received, under the will of the late Professor David Hughes, a legacy of upwards of £90,000. Professor Hughes left an equal sum to Charing Cross Hospital, King's Hospital, and Middlesex Hospital. By the terms of the will the hospital would not get the full benefit of the bequest for 21 years. A portrait had been put up in the board-room with a suitable inscription. The number of in-patients during the quarter ended June 4th was 3,001, of whom 1,137 were discharged cured, 1,550 relieved, while 314 died. The number of patients in the wards was 660, being exactly the number of the daily average. The out-patients numbered 14,130, dental patients and minor casualties, 35,851. Since the last court in March the annual report and accounts for 1899 had been printed, and now lay on the table. The receipts were: Ordinary, £61,521; extraordinary, £32,158 (including £5,000 from the Prince of Wales's Fund). The ordinary expenditure was £72,629, and the extraordinary (chiefly improvement and extension of the hospital) £21,946. Annual subscriptions amounted to £13,599, as compared with £11,020 in the previous year. The average cost of each out-patient was estimated at 1s. 1½d., as against 1s. 3⅝d. in 1898. The average stay of each in-patient worked out at 19·03 days, as contrasted with 21·77 in the previous year, and 22·18 in 1897. The total number admitted was 12,603, as compared with 11,009 in 1898. The average number of beds occupied each day was 659. The cost of each bed was £94 0s. 9d., an increase of £5 6s. The cost of each in-patient was less, viz., £4 18s. 4d., as against £5 5s. 9d. in 1898. The system in the out-patient department of charging 3d. to those who could pay it for medicines or dressings supplied was in full work throughout the year, and the amount so realised was £1,385. The total attendances amounted to 186,675; of these 108,807 paid, 12,830 being returned as having no means. Children not over school age and those recommended by doctors as request cases are not asked to pay. From the People's Subscription Fund in 1899 the total received was £1,571, as against £2,281 in the previous year. This serious falling off of £700 was attributed to the abolishing of subscribers' letters and to the charge for medicines and bandages. Both these changes had caused a certain amount of irritation, but the committee felt sure they were taking the right course, and hoped by degrees that former friends and helpers would see that the doing away of the letters was a great mercy to many suffering people who were now able to come at once to the hospital without the delay and uncertainty of getting a letter. It was also hoped that they would see it was not unfair to ask, only from those who could afford it, a small contribution toward the actual out-of-pocket expenses incurred in supplying patients with medicines and bandages. This matter was dealt with fully in the report, which also recounted the steps being taken in regard to alterations and extensions, and which have been referred to in the regular quarterly reports.

In moving the adoption of the report the Chairman alluded to the falling off in the working men's subscriptions as a matter which had caused the committee much pain and much disappointment. He thought it was a very cruel return for what was being done for them. Last year 13,000 of them or their families had been treated in the hospital, and this attitude was a little heartbreaking. It was the more curious because at the Poplar Hospital, only two miles away, with which he was also connected, and where they were doing the same thing, the working-class subscriptions had more than doubled within the last few years. Surely there could be no question that they were doing more good for poor people when the passport to help was only, Are you ill or injured? and not, Have you a letter? But apart from the fact that it took all the pleasure out of the work they would make no difference; they would still be treated with every attention and every courtesy, and he felt sure that when the soreness had been forgotten a more generous feeling would prevail.

Mr. Loftus seconded the motion, and endorsed the chairman's regret at the attitude of some of the working men's societies.

The resolution was adopted, and some formal resolutions terminated the proceedings.

